# Function of Biohydrogen Metabolism and Related Microbial Communities in Environmental Bioremediation

**DOI:** 10.3389/fmicb.2019.00106

**Published:** 2019-02-14

**Authors:** Ying Teng, Yongfeng Xu, Xiaomi Wang, Peter Christie

**Affiliations:** ^1^Key Laboratory of Soil Environment and Pollution Remediation, Institute of Soil Science, Chinese Academy of Sciences, Nanjing, China; ^2^College of Resources and Environment, University of Chinese Academy of Sciences, Beijing, China

**Keywords:** bioremediation, hydrogenase, H_2_ consumption, H_2_ metabolism, H_2_ production

## Abstract

Hydrogen (H_2_) metabolism has attracted considerable interest because the activities of H_2_-producing and consuming microbes shape the global H_2_ cycle and may have vital relationships with the global cycling of other elements. There are many pathways of microbial H_2_ emission and consumption which may affect the structure and function of microbial communities. A wide range of microbial groups employ H_2_ as an electron donor to catalyze the reduction of pollutants such as organohalides, azo compounds, and trace metals. Syntrophy coupled mutualistic interaction between H_2_-producing and H_2_-consuming microorganisms can transfer H_2_ and be accompanied by the removal of toxic compounds. Moreover, hydrogenases have been gradually recognized to have a key role in the progress of pollutant degradation. This paper reviews recent advances in elucidating role of H_2_ metabolism involved in syntrophy and hydrogenases in environmental bioremediation. Further investigations should focus on the application of bioenergy in bioremediation to make microbiological H_2_ metabolism a promising remediation strategy.

## Introduction

It is well established that the main sources of molecular hydrogen (H_2_) are geochemical and anthropogenic activities and the main sink is the biological consumption of H_2_ in soil ecosystems. The H_2_ cycle can influence air quality and climate indirectly via effects on the oxidative capacity of the atmosphere (Ehhalt and Rohrer, 2009). In addition, the H_2_ cycle plays an important role in microbial metabolism due to numerous microbial processes that depend on H_2_ production and consumption (Vignais and Billoud, 2007; Greening et al., 2015b). For example, most of the tropospheric H_2_ is consumed by soils due to the capacity of the majority of H_2_-oxidizing bacteria displaying high affinity for H_2_ in soils to recycle it (Constant et al., 2010). H_2_ is also a key metabolic compound in many anoxic ecosystems and its oxidation may support deep subsurface lithoautotrophic microbial ecosystems (Chivian et al., 2008; Nyyssonen et al., 2014; Wu et al., 2015; Bagnoud et al., 2016). The activities of H_2_-producing and consuming microbes therefore shape the global H_2_ cycle and may have vital relationships with the global cycling of other elements including carbon, sulfur, and nitrogen.

The first H_2_-oxidizing microorganisms were discovered in the 1900s (Kaserer, 1905; Stephenson and Stickland, 1931). The physical properties of H_2_ (e.g., its diffusion coefficient, 4 × 10^-9^ m^2^ s^-1^, and redox potential, E^0^’ = -0.42 V, make it relatively active in biological processes (Greening et al., 2016). H_2_ has dual physiological functions in microorganisms. Firstly, microbial fermentation of H_2_ produced by facultative or obligate fermenters can disperse excess reductant from fermentative metabolism, for example in *Escherichia coli* and *Clostridium* spp. (Trchounian et al., 2012, 2017a,b). Secondly, prokaryotic microorganisms with different metabolic processes such as hydrogen-oxidizing bacteria, methanogens and anoxygenic phototrophic bacteria can exploit H_2_ as an energy source and reductant (Schwartz et al., 2013). There are also a wide range of microorganisms with the ability to metabolize H_2_ such as aerobes and anaerobes and lithotrophs and phototrophs (Vignais and Billoud, 2007; Schwartz et al., 2013; Peters et al., 2015; Greening et al., 2016). Furthermore, recent studies show that some aerobic soil acidobacteria and actinobacteria can exploit low levels of H_2_ for survival in addition to growth, which challenges the traditional belief that H_2_ metabolism is restricted to high-H_2_ and low-O_2_ environments (Constant et al., 2010; Osborne et al., 2010; Greening et al., 2014, 2015a,b; Liot and Constant, 2016).

Hydrogenases catalyze microbial H_2_ production and consumption and are reversible enzymes responsible for reversible or partial catalytic reactions as follows (Equation 1).

(1) $$H_{2}\Leftrightarrow H^{-}+H^{+}\Leftrightarrow 2H^{+}+2e^{-}$$

On the basis of the metal cofactors of their H_2_-binding sites, these hydrogenases can be divided into three categories, namely the [NiFe]-, [FeFe]-, and [Fe]-hydrogenases (Vignais and Billoud, 2007; Schilter et al., 2016). [NiFe]-hydrogenases are closely related to both H_2_ production and consumption, while [FeFe]-hydrogenases are responsible mainly for the production of H_2_ owing to their higher turnover rate and activity compared with [NiFe]-hydrogenases (Marshall et al., 2012). However, [Fe]-hydrogenases have so far only been found in methanogenic archaea without cytochromes (Thauer et al., 2010). A recent study shows that the three different types of hydrogenase contain many subgroups based on the properties of metalloenzymes (such as metal-binding motifs, amino acid sequence phylogeny, reported biochemical characteristics and predicted genetic organization), and hydrogenase-encoding genes have also been identified in many microorganisms indicating a broad ecological distribution (Trchounian et al., 2011; Greening et al., 2016). Although the contribution of H_2_ metabolism to the entire ecosystem function is recognized in several environments such as hydrothermal vents, anoxic sediments and animal guts (Vignais and Billoud, 2007; Schwartz et al., 2013; Greening et al., 2016), the functions of hydrogenases in ecosystems in general remain largely unknown.

H_2_ metabolism plays a vital role in stability and performance in many microbial biotopes at ecosystem level (Marshall et al., 2012). It has been gradually recognized that hydrogenases may be used in bioremediation (Vignais and Billoud, 2007; Jugder et al., 2013). Numerous studies have shown that H_2_ can be utilized as an electron donor for reductive dehalogenation by many microorganisms and the occurrence of hydrogenases involved has been reported in dehalogenated bacteria (Seshadri et al., 2005; Rahm et al., 2006; Vignais and Billoud, 2007). In addition, microbial hydrogenases have been used in the remediation of metal-containing industrial wastes for the reduction of potentially toxic metals (Li et al., 2018). Under the impact of hydrogenases, microbial metabolic activities can influence the cycling of belowground minerals and organic matter and play a positive role in the bioremediation of both organic and inorganic pollutants (Lovley, 1993, 2008; Lovley and Coates, 2000; Vignais and Billoud, 2007). Thus, the use of hydrogenases for the remediation of polluted soils might be a promising strategy. In this review we attempt to integrate our understanding of the role of H_2_ metabolism in environment and environmental bioremediation processes and summarize the knowledge of H_2_ metabolism and hydrogenases involved in bioremediation.

## Microbial H_2_-Producing Processes and Their Impact on the Environment

### Fermentative Hydrogen Production From Organic Compounds

**FIGURE 1:**
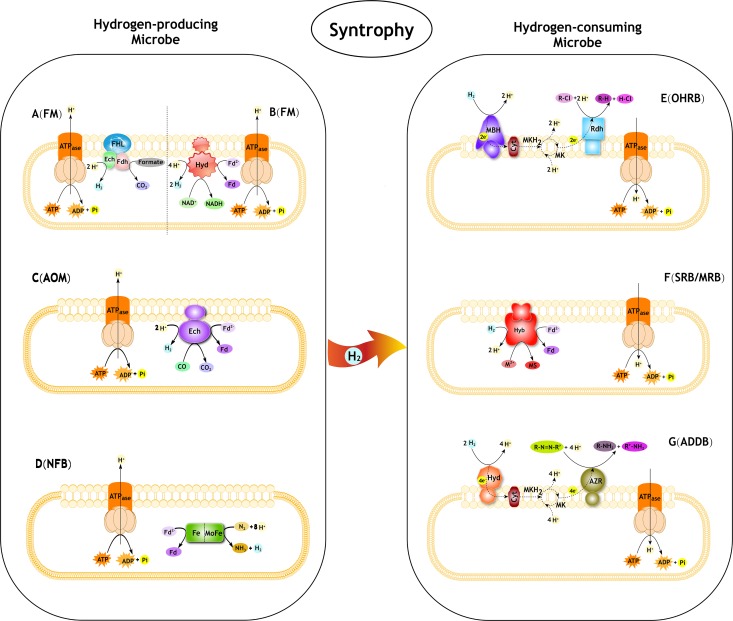
Syntrophic interactions between hydrogen-producing and hydrogen-consuming microbes in pollutant degradation. FM, Fermentative microbe; AOM, Anaerobic CO oxidation microbe; NFB, Nitrogen-fixing bacteria; OHRB, Organohalide-respiring bacteria; SRB, Sulfate-reducing bacteria; MRB, Metal-reducing bacteria; ADDB, Azo dyes decolorization bacteria. **(A)** The progress of formate oxidation coupled to H_2_ formation in *E. coli* (derived from Sawers, 1994; Hallenbeck, 2009; Trchounian et al., 2012). The FHL complex consist of a Ech type membrane-bound H_2_-evolving [NiFe] hydrogenase coupled to a formate dehydrogenase (Fdh) and membrane integral subunits. **(B)** The progress of re-oxidation of NADH coupled to H_2_ formation in *T. roseopersicina* (derived from Jenney and Adams, 2008; Maróti et al., 2010). The Hyd is a membrane-bound H_2_-evolving [FeFe] hydrogenase. Fd, ferredoxin. **(C)** The progress of anaerobic CO oxidation coupled to H_2_ formation in *C. hydrogenoformans* (derived from Svetlichny et al., 1991; Soboh et al., 2002). The Ech is a membrane-bound H_2_-evolving [NiFe] hydrogenase. Fd, ferredoxin. **(D)** The progress of producing H_2_ as a byproduct of N_2_ fixation. The nitrogenase complex consist of a Fe protein and MoFe protein. **(E)** The role of H_2_ in reductive dechlorination in *Dehalococcoides* spp. (derived from Jugder et al., 2016). MBH, membrane-bound uptake hydrogenase. Cyt, cytochrome. MK, menaquinone; MKH_2_, dihydromenaquinone. Rdh, reductive dehalogenase. R-Cl, organohalide. **(F)** The role of H_2_ in reductive PTEs in *Desulfovibrio fructosovorans* (derived from Chardin et al., 2003; Cao et al., 2014). Hyb is a membrane-bound H_2_-uptake [NiFe] hydrogenase. Fd, ferredoxin. M, PTEs. MS, metal sulfides. **(G)** The role of H_2_ in reductive azo compounds in *Shewanella decolorationis* (derived from Hong et al., 2007, 2008). Hya, membrane-bound uptake [NiFe]-hydrogenase. Cyt, cytochrome. MK, menaquinone; MKH_2_, dihydromenaquinone. AZR, azo reductase.R-N = N-R’, azo compounds.

H_2_ is a key compound in the metabolism of many anaerobes, as well as a few aerobes, which owed the capacity to use this energy-rich molecule when it is available in the environment and derive electrons from its oxidation to drive energy generation. In the absence of external electron acceptors, many anaerobic bacteria can exploit carbohydrate rich substrates to produce H_2_ by reducing protons continuously. As described previously (Das and Veziroǧlu, 2001; Das and Veziroglu, 2008; Hallenbeck, 2009), the fermentative process generating H_2_ comprises two major pathways. In the first, butane 2,3 diol fermentation or mixed acid fermentation produces H_2_ via formate decomposition where glucose is transformed to pyruvate and then releases electrons to produce H_2_ under hydrogenase through a series of oxidation and reduction reactions (Figure 1A). The second is an NADH pathway in which H_2_ is produced by the re-oxidation of NADH (Figure 1B). In the various pyruvate metabolic pathways, H_2_ is usually produced by butyric acid fermentation, mixed acid fermentation, and bacterial ethanol fermentation (Ren et al., 2005). Fermentative microorganisms such as *Clostridium* spp. (e.g., *C. butyricum* and *C. acetobutylicum*) (Fang et al., 2006; Zhang et al., 2006), rumen flora (e.g., *Butyrivibrio fibrisolvens, Eubacterium limosum, Megasphaera elsdenii, Ruminococcus flavefaciens*, and *Ruminococcus albus*) (Miller and Wolin, 1973; Joyner et al., 1977; Miller and Wolin, 1979; Chaucheyras-Durand et al., 2010), *Enterobacter* spp. (e.g., *E. cloacae* and *E. aerogenes*) (Kumar and Das, 2000; Fabiano and Perego, 2002), *Pyrococcus furiosus* and *Thermococcus litoralis* (Malik et al., 1989; Rákhely et al., 1999; Schwartz et al., 2013) have been found to effectively produce H_2_ via different pyruvate metabolic pathways. Hydrogenase enzymes also play an important role in fermentative H_2_ production (Woodward et al., 2000; Trchounian et al., 2012). In general, H_2_ production could be catalyzed by a soluble [FeFe]-hydrogenase or a special class of membrane bound [NiFe]-hydrogenase (Ech). For example, *Escherichia coli* can transform intermediary fermentation products to the gaseous products H_2_ and CO_2_ by formate hydrogenlyase reaction (Figure 1A) (Sawers, 1994). Soboh et al. (2004) report that a ferredoxin-dependent [NiFe]-hydrogenase and a NADH-dependent [Fe]-hydrogenase may catalyze H_2_ evolution from NADH in *Thermoanaerobacter tengcongensis*. Production of H_2_ by fermentation in *Thermotoga maritima* is catalyzed by a heterotrimeric [FeFe]-hydrogenase and two cytoplasmic [NiFe]-hydrogenases have been identified in *Thiocapsa roseopersicina* (Figure 1B) (Jenney and Adams, 2008; Maróti et al., 2010).

### Anaerobic Carbonic Monoxide (CO) Oxidation

There are several microbes owing different types of hydrogenogens that grow anaerobically in the dark and can unitize CO as the sole energy source to produce H_2_ (Figure 1C). Uffen (1976) and Fox et al. (1996a,b) showed that *Rhodospirillum rubrum* can produce H_2_ by oxidation of CO with the reduction of protons under the catalysis of a complex enzyme consisting of a CO-insensitive [NiFe]-hydrogenase and carbon monoxide dehydrogenase. A typical example of this group is the strictly anaerobic *Carboxydothermus hydrogenoformans* which contains a multienzyme membrane-bound [NiFe]-hydrogenase (Ech) complex (Figure 1C) (Svetlichny et al., 1991; Soboh et al., 2002). These enzymes together can oxidize CO and subsequently reduce the protons derived from H_2_O to form molecular H_2_. *Carboxydocella thermautotrophica* (Sokolova et al., 2002), *Thermosinus carboxydivorans* (Sokolova et al., 2004), *Thermincola carboxydiphila* (Sokolova et al., 2005), and *Thermolithobacter carboxydivorans* (Sokolova et al., 2007) are also thermophilic hydrogenogens.

### Production of H_2_ as a Byproduct of N_2_ Fixation

Nitrogen fixation is one of the main processes of biogenic H_2_ production and is catalyzed by nitrogenase (Figure 1D). Approximately 30–50% of the entire reduction power consumed by nitrogenase is laterally tracked to H_2_ evolution (Brewin, 1984; Evans et al., 1987). However, H_2_ is not both a competitive inhibitor of N_2_ fixation and also represents a net loss of energy unless the H_2_ can be reprocessed by means of the uptake hydrogenase (Kosourov et al., 2014). Many H_2_-utilizing microorganisms such as the aerobic H_2_-oxidizing bacteria in soils reduce the loss of energy (Stein et al., 2005; Maimaiti et al., 2007; Constant et al., 2008; Osborne et al., 2010; Annan et al., 2012; Greening et al., 2015b). Many rhizobia can symbiotically fix dinitrogen in the root nodules of legumes and produce H_2_ concomitantly. The most-studied symbiotic nitrogen-fixing bacteria in legumes include *Bradyrhizobium japonicum, Mesorhizobium mediterraneum, Sinorhizobium meliloti*, and *Rhizobium leguminosarum* (Nour et al., 1995; Spaink, 2000; Laranjo et al., 2014). In addition, strains of *Azotobacter* (Cocking, 2003) and various cyanobacteria (e.g., *Anabaena cylindrica, Nostoc muscorum*, and *Westiellopsis prolica*) (Bulen et al., 1965; Fay, 1992; Nandi and Sengupta, 1998; Das and Veziroǧlu, 2001; Das and Veziroglu, 2008) can produce hydrogen through the nitrogen fixation process. Gest and Kamen (1949) report that *Rhodospirillum rubrum* can evolve significant amounts of H_2_ in the light and this is termed the photoproduction of H_2_ caused by nitrogenase-catalyzed reduction of protons (Bulen et al., 1965). Photoproduction of H_2_ was subsequently discovered in other phototrophic bacteria such as *Rhodobater capsulatus, Rhodobater sphaeroides, Rhodobater palustris, Thiocapsa roseopersicina*, and *Halobacterium halobium* (Vincenzini et al., 1982; Gogotov et al., 1991; Khan and Bhatt, 1991; Krahn et al., 1996; Fascetti et al., 1998).

### Effects of Microbial Hydrogen Production on Environment

Atmospheric H_2_ is derived mainly from anthropogenic activities and oxidation of atmospheric methane (CH_4_) and non-methane hydrocarbons. An H_2_ mixing ratio of 0.53 ppmv is typically found in the global atmosphere (Novelli et al., 1999) and participates in atmospheric chemical cycles of H_2_O and greenhouse gasses as well as various pollutants (Schlegel et al., 1976; Crutzen and Fishman, 1977; Salvi and Subramanian, 2015; Talibi et al., 2017). In addition, H_2_ is a potential future energy carrier that may significantly affect the atmospheric H_2_ budget when used on a large scale (Brenninkmeijer et al., 2003; Petersen et al., 2011). It has been estimated that the total amount of H_2_ emissions into the troposphere each year is approximately 107 Tg (Rhee et al., 2006). Tromp et al. (2003) reported that the concentrations of stratospheric H_2_O and ozone and stratospheric temperatures would be affected by these H_2_ emissions. Moreover, the potential impacts of an increase in anthropogenic H_2_ emissions on the concentration of other trace gasses such as CH_4_ and CO) are also proposed.

About 7–11% of the global H_2_ pool is contributed by all oceanic, lake, and soil organisms (Schwartz et al., 2013). ‘Hot spots’ can be found in hypersaline cyanobacterial mats, with the release of H_2_ concentrations between 16,000 and 90,000 ppmv (Nielsen et al., 2015), which might be the main source of H_2_ emission from lake surface waters to the atmosphere. Numerous studies show that both cell counts of cyanobacteria and their N_2_ fixation rates are correlated with the H_2_ concentration of lake water (Conrad et al., 1983; Schütz et al., 1988; Schmidt and Conrad, 1993). Furthermore, the production of fermentation H_2_ and organic acids is a key component in the biogeochemistry of microbial mats, which promotes close interactions between anoxygenic phototrophs, cyanobacteria and heterotrophic bacteria (Otaki et al., 2012; Lee et al., 2014; Nielsen et al., 2015). However, almost all of the H_2_ produced from hypoxic sediments is also consumed by the sediments (Schwartz et al., 2013). The effects of hydrogen consumption on microbial communities in sediments therefore deserve further study.

The contribution of soils to the atmospheric H_2_ reservoir is more complex because soils are the main sink of the global H_2_ cycle, accounting for about 75 to 80% of atmospheric absorption (Constant et al., 2009; Ehhalt and Rohrer, 2009). However, nitrogen-fixing bacteria that form symbioses with legumes or free-living N_2_ fixing bacteria can generate large amounts of H_2_ as a by-product during N_2_ fixation (Orr et al., 2011; Mus et al., 2016). It has been estimated that H_2_ concentrations inside N_2_-fixing legume nodules range from 9,000 to 27,000 ppmv (Hunt et al., 1988; Witty, 1991; Witty and Minchin, 1998), so that diffusion losses during the growing season might reach 240,000 L H_2_ (Dong et al., 2003). Thus, the intensity of these H_2_ emissions to soils is determined by the hydrogen-metabolic capabilities of rhizobacterial symbionts (*Hup*^+^ or *Hup*^-^ genotypes) in nodules through an uptake [NiFe]-hydrogenase (Evans et al., 1988; Annan et al., 2012). In the *Hup*^+^ legume rhizosphere the energy of H_2_ can be recycled by the [NiFe]-hydrogenase, while H_2_ is released into the surrounding soil in the *Hup*^-^ legume rhizosphere. There is thus increasing evidence that H_2_ released into surrounding soils plays a key role in increasing plant biomass via the enrichment of aerobic H_2_-oxidizing bacteria (HOB), or plant growth-promoting rhizobacteria (PGPR) in both legumes and non-legumes (Dong et al., 2003; Maimaiti et al., 2007). Different H_2_ mixing ratios found in natural ecosystems may indeed lead to changes in soil microbial community structure and coordinated feedback of community functions. Constant et al. (2008) found that soil actinomycetes (such as *Streptomyces* sp. PCB7) are the main users of trace level of H_2_ in soils and might be key contributors to the function of soils as a sink in the global H_2_ cycle. Subsequently, Khdhiri et al. (2017) validated their own hypothesis by showing that the taxonomic response of the soil microbial community composition to H_2_ exposure is inconsistent across land use types. Piché-Choquette et al. (2018) revealed that H_2_ supports metabolic and energetic flexibility in microorganisms supplying a variety of ecosystem services via dose-response relationships between environmentally relevant H_2_ concentrations and the biological sinks of H_2_, CH_4_, and CO in soils.

## Role of H_2_ in Environmental Bioremediation

The H_2_ produced both biogenically and abiogenically can be released and provided to support for the growth and metabolism of hydrogenotrophic prokaryotes (Karyakin et al., 2007). H_2_ metabolism fulfills a critical role in the ecosystems of many microbial biotopes (Vignais and Billoud, 2007; Schwartz et al., 2013; Greening et al., 2016). It is currently considered that a wide range of microbial groups employ H_2_ as an electron donor to catalyze the reduction of pollutants such as organohalides, azo compounds and potentially toxic elements.

### Organohalides

Organohalides are recalcitrant, toxic, highly persistent, globally prevalent, and carcinogenic environmental contaminants. Organohalide-respiring bacteria (OHRB) have been isolated from polluted soils, sludges, sediments, aquifers, freshwaters, and marine habitats, and they are of considerable importance in bioremediation processes and natural halogen cycles (Zanaroli et al., 2015). Most OHRB discovered to date belong to *Desulfomonile, Dehalococcoides, Dehalobacter, Desulfitobacterium, Desulfuromonas*, and *Sulfurospirillum* (formerly *Dehalospirillum*) as reviewed by Jugder et al. (2015). Reductive dechlorination is an anaerobic respiration process that utilizes H_2_ as electron donor to dehalorespire these halogenated organics (Figure 1E) (Zanaroli et al., 2015; Agarwal et al., 2017). The process of electron via electron transport phosphorylation from the oxidation of the H_2_ to reductive dechlorination of organohalides involving membrane associated oxidoreductases (Figure 1E) (Jugder et al., 2016). Membrane-bound hydrogenases (MBH) are the initial oxidizers to take up the electrons released from molecular H_2_, which play a vital role in organohalide respiration (Jugder et al., 2013, 2015, 2016). The reductive dehalogenation of organohalides is typically catalyzed by dehalogenating enzyme systems coupled to ATP synthesis, reductive dehalogenases (Rdases) replace the halogen substituent with a hydrogen atom, reducing the toxicity and recalcitrance to biodegradation (Figure 1E) (Adrian and Loeffler, 2016; Gevorgyan et al., 2018). Sequencing data of genomes reveal that OHRB possess as many as 36 putative Rdases. After transformation to lower halogenated organics under anaerobic conditions, these toxic compounds can subsequently be mineralized by aerobic bacteria (Jugder et al., 2015).*Desulfomonile tiedjei* strain DCB-1 is one of the best-described dechlorinating anaerobes. The strain, first discovered by Suflita et al. (1982), reductively dechlorinates 3-chlorobenzoate while replacing the chlorine atom with hydrogen from H_2_, whereby providing energy for bacterial growth (Shelton and Tiedje, 1984; Dolfing and Tiedje, 1986; Dolfing and Tiedje, 1987). The strain was then noted to consume H_2_ with 3-chloro-, 3-bromo-, 3-iodobenzoate, tetrachloroethene (PCE), trichloroethene (TCE) (Cole et al., 1995), and chlorophenols (Mohn and Kennedy, 1992) as electron acceptors (DeWeerd et al., 1991). During the dehalogenation of 3-chlorobenzoate, formate was the most effective electron donor, followed by H_2_, pyruvate, and acetate.

*Dehalococcoides* strains are also some of the best known species capable of reductively dechlorinating a wide range of haloorganics including chlorinated benzenes, biphenyls, dioxins, ethenes, naphthalenes, and brominated diphenyl ethers. For example, tetrachloroethene is a commonly used solvent that possesses high toxicity and is a suspect carcinogen. The complete reductive dechlorination of tetrachloroethylene (PCE) and trichloroethylene (TCE) to non-toxic ethylene was first observed under methanogenic conditions by mixed cultures (Freedman and Gossett, 1989). Although H_2_ also served as the electron donor, methanol was more effective in sustaining the reductive dechlorination process. Holliger et al. (1993) isolated an anaerobic bacterial culture, previously named as PER-K23, from an anaerobic packed-bed column. By using H_2_ and formate as the only electron donors, PCE or TCE was reductively transformed to ethane via *cis*-1,2-dichloroethene (*cis*-1,2-DCE), chloroethene, and ethene, which was coupled to bacterial growth. The key role of hydrogenases in metabolizing these pollutants is underscored by the fact that both uptake (Hup type) and energy-conserving hydrogenases (Hyc or Ech type) were found in the genome of *Dehalobacter restrictus* PER-K23 (Rupakula et al., 2013). Maymó-Gatell et al. (1997) then isolated a dehalogenator, strain 195, and characterized it as *Dehalococcoides ethenogenes*. To date, *Dehalococcoides* species are the only bacteria known to be capable of completely dechlorinating chloroethylene. Genomic analysis of *Dehalococcoides ethenogenes* 195 showed that several hydrogenase genes including the membrane-bound periplasmic Hup, cytoplasmic Vhu, and membrane-bound Ech and Hyc [NiFe]-hydrogenases (Groups 1, 3, 4, and 4, respectively), and a membrane-bound Hym [Fe]-hydrogenases has potential roles in electron transport, which are capable of completing anaerobic dechlorination of the solvents PCE and TCE to vinyl chloride (VC) and ethane (Vignais et al., 2001; Morris et al., 2006).

Unlike other halorespiring bacteria, *Dehalococcoides* spp. use only H_2_ as an obligate electron donor for the dechlorination reaction, and no other electron acceptors support growth. For example, *D. ethenogenes* strain 195 grew only on H_2_ as electron donor for both bacterial growth and PCE reduction rather than formate, lactate, methanol, ethanol, glucose, pyruvate, or yeast extract (Maymó-Gatell et al., 1997). In addition, *Dehalococcoides* sp. CBDB1 was the first purified isolate of a bacterium relying on the energy obtained from stoichiometrical dehalorespiration of chlorobenzenes (CB) such as 1,2,3-trichlorobenzene (TCB) and 1,2,3,4-tetrachlorobenzene (TeCB) (Adrian et al., 2000). Both *Dehalococcoides* sp. 195 and CBDB1 exhibit reductive dehalogenation of chlorophenols (Adrian et al., 2007). Kube et al. (2005) compared the genome sequence of *Dehalococcoides* sp. CBDB1 with *Dehalococcoides ethenogenes* strain 195 and revealed that the hydrogenases previously described for strain 195 are also present in strain CBDB1. Chloroform (CF, CHCl_3_) is a non-polar solvent that is ubiquitous and is toxic to humans. The biodegradation of CF involves two processes, (1) dehalorespiration in which CF is dechlorinated to dichloromethane (DCM, CH_2_Cl_2_) by employing H_2_ as electron donor under the action of uptake hydrogenase, and (2) a fermentative process in which DCM is transformed to H_2_, acetate and carbon dioxide. Lee et al. (2012) report the involvement of *Dehalobacter* in dehalorespiration of CF [Equation (2)].

(2) $${\mathrm{CHCl}}_{3}+H_{2}\to {\mathrm{CH}}_{2}{\mathrm{Cl}}_{2}{\mathrm{+H}}^{+}+{\mathrm{Cl}}^{-}$$

Despite these findings in respiration of organohalides, there is no real consensus on the involvement of various membrane associated components.

### Potentially Toxic Elements (PTEs)

Potentially toxic elements display environmental durability, biological accumulation, and potential biological toxicity. The remediation of PTEs can be achieved by sulfate-reducing bacteria (SRB) or metal-reducing bacteria that can utilize H_2_ or other organic compounds as terminal electron donors to reduce the PTEs. Tebo and Obraztsova (1998) isolated the first sulfate-reducing bacterium from PTE-polluted sediments named *Desulfotomaculum reducens* sp. nov. strain MI-1, which can utilize H_2_ as terminal electron donor and metals [such as Cr(VI), Mn(IV), Fe(III), and U(VI)] as electron acceptors accompanied by bacterial growth. Thus far, more than 40 SRB species have been identified, including *Desulfobacter, Desulfovibrio, Desulfotomaculum* and *Desulfomicrobium*, and others (Leloup et al., 2009; Mizuno et al., 2012; Hussain et al., 2016; Li et al., 2018). Subsequently, due to the advantages of SRB (no secondary pollution and strong adaptability), they have been used in the bioremediation of PTEs (Li et al., 2018). Generally speaking, there are two steps involved in the mechanism of SRB removal of PTEs from wastewaters: (i) SRB utilize sulfate as electron acceptor to oxidize simple organic compounds to generate bicarbonate ion and hydrogen sulfide under anaerobic conditions [Equation (3)], and (ii) the hydrogen sulfide generated reacts with dissolved PTE to form insoluble metal sulfide precipitates [Equation (4)] (Kieu et al., 2011; Singh et al., 2011; Li et al., 2017).

(3) $${\mathrm{2CH}}_{2}O+{\mathrm{SO}}_{4}^{2-}\to 2{\mathrm{HCO}}_{3}^{-}+H_{2}S$$

(4) $$H_{2}S+M^{2+}\to 2H^{+}+\mathrm{MS}(\mathrm{S)}$$

Where CH_2_O represents simple organic compounds (such as acetate and lactate), M represents PTEs, and MS represents metal sulfides. Because of their special characteristics with the corresponding metal sulfides readily forming precipitates, SRBs have been used to treat PTE-polluted wastewaters (e.g., uranium-containing, chromium-containing and antimony-containing wastewaters, organochlorines, and other pollutants) (Li et al., 2018). Lovley and Phillips (1994) showed that the bioremediation effect of *Desulfovibrio vulgaris* which utilizes H_2_ as the electron donor catalyzed by the *c*_3_ cytochrome functions as a Cr(VI) reductase in Cr(VI)-contaminated waters was superior to the previously described Cr(VI) reductive microorganisms. Kieu et al. (2011) reported that the PTE removal efficiencies of Cu^2+^, Ni^2+^, Zn^2+^, and Cr^6+^ in anaerobic semi-continuous stirred tank reactors containing a consortium of SRB reached 94–100% after 4 weeks under experimental conditions. In addition, several microbial genera reduced uranium to form easily precipitated reduced U(IV) species, and this has been used successfully in soil remediation (Phillips et al., 1995; Fredrickson et al., 2000; Valls and De Lorenzo, 2002).

Several uptake hydrogenases were considered to have potential application in the bioremediation of PTEs. The [NiFe] uptake hydrogenases in group 1 including membrane-bound respiratory uptake hydrogenases that couple H_2_ oxidation to catalyze metal reduction (Figure 1F). For example, [NiFe]-uptake hydrogenase from SRB can reduce toxic chromate VI to form a less toxic product (Chardin et al., 2003). In addition, technetium VII is reduced by *Desulfovibrio fructosovorans* through this mechanism (Tabak et al., 2005), and hydrogenases involving in metal reduction have also been observed in other metals including ferrum (Fe) (Coppi et al., 2004), platinum (Riddin et al., 2009), and lead (Deplanche et al., 2010). A comprehensive analysis of the genome sequence of the metal-reducing bacterium (*Shewanella oneidensis*) has been conducted, and has predicted that an [Fe]-hydrogenase and several cytochromes are involved in the electron transport and metal reduction processes (Heidelberg et al., 2002). However, the potential application of microbes with different subgroup hydrogenases for PTE respiration is not enough, requiring further study including the biochemical investigations of these different subgroup hydrogenases.

### Other Pollutants

Azo compounds undergo dissimilatory azoreduction by *Shewanella decolorationis* S12 under anaerobic conditions. This strain utilized azo compounds as carbon source for growth by azo reductase which is sustained by the H_2_ supply. The strain also catalyzed H_2_-dependent reduction of Fe(III) and humic substances (Coppi et al., 2004; Hong et al., 2008). Brigé et al. (2008) show that *Shewanella decolorationis* MR-1 utilized azo dye amaranth as electron acceptor for microbial energy conservation. Mutambanengwe et al. (2007) show the decolorization of a wide range of azo dyes with sulfate-reducing microbes (SRM) and hydrogenases might be involved in the degradation process. A multicomponent electron transfer chain has been proposed to be involved in the extracellular reduction of azo compounds. The electron transfer components consist of the cytoplasm/outer membrane, periplasm, c-type cytochromes, and menaquinone (Hong et al., 2007; Brigé et al., 2008). Hya type [NiFe]-hydrogenase or Hyd type [Fe]- hydrogenase act as a critical hub mediating the oxidization of H_2_ to provide electrons for azoreduction metabolism (Figure 1G) (Hong et al., 2008).

H_2_-dependent reduction has been reported in nitroaromatic compounds (Watrous et al., 2003). In a strict anaerobe, *Clostridium acetobotulinicum*, an [Fe]-hydrogenase is responsible for the reduction of nitro substituents of 2,4,6-trinitrotoluene (TNT) to the corresponding hydroxylamine in an acidogenic environment.

### Factors Affecting the Utilization of Hydrogen by Degrading Bacteria in the Environment

There are many factors affecting the utilization of H_2_ by degrading bacteria in the environment such as H_2_ source, H_2_ transfer process and other environmental factors (including trophic hierarchies, external pH, osmotic coditions, concentration of carbon sources and their mixtures and microbial community and other physicochemical factors).

Methanogens were found to affect the interspecies H_2_ transfer of dehalorespiring bacteria, which might promote or inhibit the dechlorination process (Smatlak et al., 1996; Fennell et al., 1997; Yang and McCarty, 1998). Johnson et al. (2008) demonstrated the dechlorination of stress-related net cell growth by *Dehalococcoides ethenogenes* strain 195 (DE195) which was isolated and then transited to a smooth phase. Although *Methanobacterium congolense* (MC) can compete with DE195 for hydrogen, adverse effects of the dechlorination rate were not observed (Men et al., 2012). This is mainly because the H_2_ threshold required for dechlorination is very low, so that even though methane production consumes a large amount of H_2_, it does not compete for dechlorination (Yang and McCarty, 1998; Men et al., 2012). In syntrophic communities, H_2_-producing bacteria and H_2_-consuming methanogens perceive the redox conditions and affect each other’s metabolism (Stams and Plugge, 2009). Several studies have shown that the reduction dechlorination can be promoted in some communities in the presence of methanogens (Vogel and McCARTY, 1985; Heimann et al., 2006; Kong et al., 2014). In addition, a recent study found that *Methylobacter* seemed to be tolerant to TCE and may play a vital role in TCE degradation (Kong et al., 2014). Although many studies have assessed the association between methanogens and dechlorination bacteria, the mechanism by which methanogens affect dechlorinating communities remains unclear.

The process of forming compact aggregates involves both physicochemical and biological interactions (Stams and Plugge, 2009). When the compact aggregates are formed in anaerobic bacteria and methanogenic archaea, the rate of H_2_ transfer between two species increases significantly (Lettinga et al., 1988; Stams and Plugge, 2009). Several studies have also shown that the inter-microbial distances affect both their specific growth rates and biodegradation rates (Ishii et al., 2005; Stams et al., 2006; Stams and Plugge, 2009). Thus, forming compact aggregates might be an important factor influencing the biodegradation rates of degrading bacteria.

It is well known that trophic hierarchies occur because different functional members of the community provide each other with a matrix and basic cofactors, and eliminate inhibitory metabolites (Schink, 1997; Rittmann and McCarty, 2012). DeWeerd et al. (1991) reported that acetylene, molybdate, selenate, and metronidazole can inhibit dehalogenation, sulfite reduction and H_2_ metabolism, indicating that the reduction of sulfite and dehalogenation may share part of the same electron transport chain. However, some environmental factors might accelerate the degradation of pollutants by promoting H_2_ utilization. For example, cobalamin has a positive effect on the dechlorination process as a co-factor of the reductive dehalogenases (Yan et al., 2012). *Desulfovibrio vulgaris* Hildenborough (DVH) possesses the full set of genes required for the biosynthesis of adenosylcobalamin, a derivative of vitamin B12 which might result in an increased concentration of the corrinoid co-factor (vitamin B12) in co-cultures, taken up and utilized immediately by *Dehalococcoides* species (Rodionov et al., 2004). In addition, the main factors influencing H_2_ utilization such as external pH, osmotic conditions, concentration of carbon sources and their mixtures, microbial community and other physicochemical factors mainly affected growth and the physiological activity including uptake hydrogenase and pollutant degrading enzymes of the degrading bacteria (Richter and Gescher, 2014; Trchounian and Trchounian, 2014, 2015; Trchounian et al., 2017a).

## Interspecies Hydrogen Transfer During Syntrophic Growth

Syntrophy coupling mutualistic interactions between hydrogen-/formate-producing and hydrogen-/formate-consuming microorganisms is essential for biofuel production, pollutant degradation, and global carbon cycling (Kleinsteuber et al., 2012; Sieber et al., 2012; Morris et al., 2013). When sulfate is limited or unavailable, SRBs can also mediate the transfer of H_2_ between species, which provides the bacterial species with a very versatile metabolism adapted to complex ecological environments. Odom and Peck (1981) first documented the transfer of the redundant H_2_ evolved from substrate fermentation by SRBs to other H_2_ consuming bacteria. Using a defined two-member continuous culture, Drzyzga et al. (2001) demonstrated that the sulfate reducer *Desulfovibrio* sp. strain SULF1 can use the dehalorespiring *Desulfitobacterium frappieri* TCE1 as a ‘biological electron acceptor’ to sustain growth. They also noted that dehalogenation of tetrachloroethene (PCE) was inhibited at sulfate concentrations above 2.5 mM, while PCE was completely dehalogenated to *cis*-dichloroethene (*cis*-DCE) with 1 mM sulfate or without sulfate addition (Drzyzga and Gottschal, 2002). In this community, *Desulfovibrio vulgaris* Hildenborough (DVH) can grow syntrophically with *Dehalococcoides ethenogenes* strain 195 (DE195), thus enhancing the robustness of bacterial growth and the dechlorination activity of trichloroethene (Men et al., 2012). The syntrophical interaction with sulfate reducers has been shown to result in more effective transfer of H_2_, thereby facilitating faster dechlorination and more rubust growth of dehalogenating strains compared with gaseous H_2_ (Men et al., 2012). The syntrophic relationship between methanogens and archaea also involves interspecies H_2_ transfer in the process of converting long-chain fatty acids (Stams and Plugge, 2009). Subsequently, Ziels et al. (2017) found several formate hydrogenases and dehydrogenases in the enriched genome bins (GBs) of both their codigesters. In the process of CF dechlorination, interspecies H_2_ transfer was observed in the form of acetogenesis and methanogenesis by Lee et al. (2012), which required syntrophic partners to maintain low H_2_ partial pressures.

The possible processes of syntrophic interactions between H_2_-producing and H_2_-consuming microbes in pollutant degradation are shown in Figure 1. Previous studies have shown that H_2_-forming bacteria and H_2_-utilizing bacteria sense redox conditions, influencing each other’s metabolism in syntrophic communities (Stams and Plugge, 2009). Interspecies electron transfer mechanisms underlie thermodynamically favorable syntrophic processes (Gieg et al., 2014). In anoxic environments, butyrate oxidations involving energy-dependent reactions were possible to be applied in syntrophic degradation of organohalides. For example, the standard free reaction enthalpies (ΔG^o^′) of butyrate oxidations and organohalide degradations were as follows [Equation (5) Müller et al., 2010; Equation (6) Jugder et al., 2016]:

(5) $${\mathrm{Butyrate}}^{-}+2H_{2}O\to {\mathrm{2Acetate}}^{-}+H^{+}+2H_{2}(\Delta G^{o^{\prime }}=+48.3KJ/mol)$$

(6) $$H_{2}+R-\mathrm{Cl}\to R-H+\mathrm{HCl}(\Delta G^{o^{\prime }}=-\;131to-192KJ/mol)$$

Based on energy balance toward H_2_ production and consumption analysis, we propose that the energy-transforming reactions between H_2_ production and organohalide degradations might be involved in syntrophic H_2_ production and consumption microorganisms. Dehalogenating microorganisms (such as *Dehalococcoides* sp. strain BAV1 and *Dehalococcoides ethenogenes* strain 195) can utilize acetate as carbon source and H_2_ as electron donor when grown in isolation, exhibiting limited dechlorination activity and low growth rates (He et al., 2003a,b). Thus, a promising method might be to develop improved bioremediation strategies by enhancing the strong growth and dechlorination activity of dehalogenating microorganisms (Men et al., 2012). However, many interspecies H_2_ transfer interactions are syntrophic, and thus only present in complex microbial communities but not in pure cultures. In complex microbial consortia, H_2_ indirectly mediates electron shuttle between electron donors and acceptors. Hydrogenotrophic bacteria can profit from the H_2_ produced from their syntrophic partners, thereby transforming pollutants. Thus, both H_2_-producing and H_2_-consuming microorganisms are essential for their own growth and might also promote the degradation of pollutants (Stams and Plugge, 2009).

## Conclusion and Perspectives

Metabolism of H_2_ including H_2_ production and H_2_ consumption have been recognized as a potential driving force affecting the structure of microbial communities and may even change community functions. Although the contribution of H_2_ metabolism to entire ecosystem processes is recognized in hydrothermal vents, anoxic sediments and animal guts (Vignais and Billoud, 2007; Schwartz et al., 2013), the role of H_2_ metabolism and hydrogenases in ecosystems are not fully elucidated. Further advances in exploiting the function of biohydrogen metabolism and related microbial communities in environmental bioremediation are expected to result from (i) using metagenome sequencing, single-gene fluorescence *in situ* hybridization, the functional gene arrays (GeoChip) and *in situ* mass spectrometry to track the dynamics of pollutant-degrading bacteria involving in H_2_ metabolism and the interplay between pollutant-degrading bacteria and H_2_-metabolism bacteria in degradation process; (ii) effects of soil conditions on H_2_-consuming microorganisms degrading pollutants; (iii) structural studies of hydrogenases or the synergistic action of other enzymes (such as ATPase and Rdase) involving in the process of environmental bioremediation and enhancing these enzymes activity through protein engineering; (iv) integrative analyses of genomic, transcriptomic, and epigenomic data in these environmental bioremediation process.

To date, environmentally friendly management techniques named “3B” techniques (biological carbon sequestration, bioenergy, and bioremediation) have been proposed to further enhance biodiversity and mitigate environmental stressors (Teng et al., 2012). Environmental H_2_ is an energy source for aerobic H_2_ oxidizers, sulfate reducers, acetogens and methanogens and is also a source of reducing power for anaerobic bacteria and anoxygenic phototrophs (Schwartz et al., 2013). Syntrophy coupling mutualistic interactions between H_2_-producing and H_2_-consuming microorganisms is not restricted to the transfer of reducing agents such as H_2_ or formate, but can also involve the exchange of organic, sulfurous and nitrogenous compounds or the removal of toxic compounds. Nevertheless, there is still a considerable need for appropriate research initiatives to apply those microbial groups to the bioremediation of contaminated soils. However, soil is a complex and dynamic biological system. From the soil to the microorganism, bioavailability of pollutants involves a full process of adsorption and desorption, transport, and uptake by microorganisms which are also affected by the soil conditions such as soil organic matter, soil minerals, soil moisture, soil aggregates and so on (Ren et al., 2018; Teng and Chen, 2019).

Proton ATPase or other membrane bound secondary transporters affect hydrogenase activity and thus H_2_ metabolism (Trchounian et al., 2011; Gevorgyan et al., 2018). So, structural studies of hydrogenases or other synergistic enzymes (such as ATPase and Rdase) involving in the process of environmental bioremediation are vital important in directing protein engineering, for example, in rendering these enzymes activity to promote the degradation efficiency of pollutants via identification of factors linked to the protein environment of the active site. Studies of H_2_ metabolism and regulation will also be important in engineering microorganisms at the cellular level to maximize the degradation efficiency of pollutants. Since hydrogenases and other synergistic enzymes have been shown to play an important role in the degradation of pollutants, it is also tempting to consider that analysis of genomic, transcriptomic, and epigenomic data of these enzymes in environmental bioremediation process will likely provide vital insights into the hydrogenase participates in degradation mechanism of pollutants.

In conclusion, this review provides a comprehensive framework for H_2_ production and H_2_ consumption in environmental bioremediation processes. The syntrophy coupling mutualistic interaction between H_2_-producing and H_2_-consuming microorganisms could be applied to the removal of toxic compounds. In addition, several uptake hydrogenases are also considered to have potential application in the bioremediation of those toxic compounds. The use of this bioenergy may provide a low-input and ecologically friendly bioremediation strategy for the future.

## Author Contributions

YT, YX, and XW collected the data. YT and YX drafted the article. YT, XW, and PC critically revised the article.

## Conflict of Interest Statement

The authors declare that the research was conducted in the absence of any commercial or financial relationships that could be construed as a potential conflict of interest.
